# Avoiding Absolute Quantification Trap: A Novel Predictive Signature of Clinical Benefit to Anti-PD-1 Immunotherapy in Non-Small Cell Lung Cancer

**DOI:** 10.3389/fimmu.2021.782106

**Published:** 2021-11-19

**Authors:** Chengming Liu, Sihui Wang, Sufei Zheng, Fei Xu, Zheng Cao, Xiaoli Feng, Yan Wang, Qi Xue, Nan Sun, Jie He

**Affiliations:** ^1^ Department of Thoracic Surgery, National Cancer Center/National Clinical Research Center for Cancer/Cancer Hospital, Chinese Academy of Medical Sciences and Peking Union Medical College, Beijing, China; ^2^ State Key Laboratory of Molecular Oncology, National Cancer Center/National Clinical Research Center for Cancer/Cancer Hospital, Chinese Academy of Medical Sciences and Peking Union Medical College, Beijing, China; ^3^ Department of Medical Oncology, National Cancer Center/National Clinical Research Center for Cancer/Cancer Hospital, Chinese Academy of Medical Sciences and Peking Union Medical College, Beijing, China; ^4^ Department of Pathology, National Cancer Center/National Clinical Research Center for Cancer/Cancer Hospital, Chinese Academy of Medical Sciences and Peking Union Medical College, Beijing, China

**Keywords:** NSCLC, immune checkpoint inhibitors, clinical benefit, prognosis, BRGPI

## Abstract

Immunotherapy has been focused on by many oncologists and researchers. While, due to technical biases of absolute quantification, few traditional biomarkers for anti-PD-1 immunotherapy have been applied in regular clinical practice of non-small cell lung cancer (NSCLC). Therefore, there is an urgent and unmet need for a feasible tool—immune to data source bias—for identifying patients who might benefit from ICIs in clinical practice. Using the strategy based on the relative ranking of gene expression levels, we herein proposed the novel BRGP index (BRGPI): four BRGPs significantly related with progression-free survival of NSCLC patients treated with anti-PD-1 immunotherapy in the multicohort analysis. Moreover, stratification and multivariate Cox regression analyses demonstrated that BRGPI was an independent prognostic factor. Notably, compared to PD-L1, BRGPI exerted the best predictive ability. Further analysis showed that the patients in the BRGPI-low and PD-L1-high subgroup derived more clinical benefits from anti-PD-1 immunotherapy. In conclusion, the prospect of applying the BRGPI to real clinical practice is promising owing to its powerful and reliable predictive value.

## Introduction

Non-small cell lung cancer (NSCLC) is related with the highest cancer-related mortality worldwide. It features a high mortality rate and only 19% of those diagnosed with NSCLC will be alive 5 years later (1, 2). Over the years, the application of molecular targeted therapy and immunotherapy has allowed many patients to survive longer (3). Although some patients can benefit from targeted molecular therapy, rapid resistance limits its effectiveness in lung cancer treatment (4). Immune-checkpoint inhibitors (ICIs)—such as pembrolizumab and nivolumab targeting PD-1—have revolutionarily improved the prognosis of patients with NSCLC. Clinical trials (5–9) and real-world data (10–12) have demonstrated that anti-PD-1/PD-L1 immunotherapy effectively improves long-term response and durable disease control. Unfortunately, only a small number of patients can derive benefit from ICIs; therefore, reliable biomarkers are needed to identify these candidate patients (13).

Biomarkers predicting immunotherapy benefits have recently emerged, including those correlated with the inflammatory tumor microenvironment, such as PD-L1 protein expression in cancer and antigen-presenting cells, and markers demonstrating the increase of tumor-specific neoantigens like tumor mutational burden (TMB) (14, 15). PD-L1 expression is the most widely recognized biomarker for ICIs targeting PD-1/PD-L1. Nevertheless, the sensitivity and specificity of this approach are modest (16). Most patients do not respond to ICIs but given high PD-L1 expression, a small group of PD-L1-low/negative patients do respond to ICIs (17). Also, due to the different antibodies and cut-off values, PD-L1 expression varies among different platforms for detection (18). Application of PD-L1 alone may be insufficient to predict the response to immunotherapy. Beyond PD-L1 expression, TMB has also been recommended as a critical marker related to the response of immunotherapy (19). Theoretically—as TMB is correlated with the number of neoantigens—the higher the TMB is, the better the immunotherapy effect will be. Yet TMB alone fails to represent the complexity of tumor immunogenicity. Anti-tumor cytotoxicity does not correlate with neoantigen load, and high TMB does not equivalent to immunogenicity and activation of anti-tumor immunity (20, 21). Like PD-L1, TMB also varies largely among different detecting platforms and there is no agreed-upon clinically validated TMB cut-off. Therefore, predictive markers—comprehensively reflecting anti-tumor immunity—are urgently needed to determine the patients who might derive benefit from anti-PD-1 immunotherapy in clinical practice, and without data platform limitations.

Opening gene expression sources in public databases enable the development of reliable gene-based biomarkers for cancer research. Some gene expression-based signatures have been proposed for diagnosis and treatment planning for patients with NSCLC. Unfortunately, few of them have been applied in regular clinical practice because of issues such as overfitting in small training datasets and insufficient validation (22, 23). Generally, adequate normalization was needed before the gene expression raw data were used, and this is difficult to accomplish owing to technical biases in different measuring platforms and sample heterogeneity among datasets. The ranking of gene relative expressions is a new approach to avoid data preprocessing, such as normalization and scaling. Methods based on this have been effective for cancer classification, immune status determination, and analyses of patients’ outcomes (24–26).

The objective of this study was to construct a predictive signature based on benefit-related gene pairs (BRGPs)—represented by four BRGPs significantly related with progression-free survival (PFS)—in NSCLC patients who received the treatment of ICIs. Considering all these decisive immune genes that may influence the response to ICIs, we constructed a predictive pattern to remedy the deficiencies of existing biomarkers.

## Materials and Methods

### Study Design and Data Collection

We enrolled 74 patients with advanced NSCLC who received the treatment of ICIs in three independent cohorts. We recruited 35 patients from GSE93157 as the signature-training dataset. We then collected 20 patients from the GSE136961 cohort and 19 patients from the CICAMS cohort for signature validation of the prognostic model. The analysis pipeline of the construction and validation of benefit-related gene-pair index (BRGPI) is shown in **Supplementary Figure S1**.

We downloaded normalized RNA-seq by expectation maximization (RSEM)-estimated count data of the GSE93157 cohort and transcripts per million (TPM) data of the GSE136961 cohort from the Gene Expression Omnibus (GEO, http://www.ncbi.nlm.nih.gov/geo), and corresponding clinical information were obtained. The CICAMS cohort included 19 LUAD patients who received the treatment of ICIs at the Cancer Hospital/Institute, Chinese Academy of Medical Sciences (CICAMS, Beijing, China) from April 2016 to July 2019. Moreover, formalin fixation paraffin embedding (FFPE) specimens of all enrolled patients prior to the initiation of ICIs were available. According to the Response Evaluation Criteria in Solid Tumors, version 1.1, the tumor response to ICIs was categorized as a complete response (CR), a partial response (PR), stable disease (SD), or progressive disease (PD). Noticeably, non-PD refers to the patients with CR, PR, or SD (27). Progression-free survival (PFS) was defined as the time from the initiation of ICIs administration to the time of PD. The Ethics Committee of CICAMS approved and oversaw this study (approval number 20/242-2438). The characteristics of individuals included in the various patient cohorts are shown in **Supplementary Table S1**.

### Construction and Validation of a Predictive Signature Based on BRGPs

We constructed BRGPI based on immune-related genes. Those immune-related genes were downloaded from the Pan-Cancer Immune Profiling Panel, including cytokines and their receptors, and genes correlated with the adaptive immune response such as antigen processing and presentation, T-cell activation, and infiltration (28). We selected 222 immune-related genes that were shared with all the cohorts to construct 2526 gene pairs for pairwise comparison. Each gene pair was scored on the basis of normalized RSEM-estimated count data of GSE93157, TPM data of GSE136961, and proteomic data of CICAMS. Noticeably, we used the immunohistochemistry (IHC) method to obtain the protein expression values of the selected BRGPs. A BRGP score was assigned on the basis of the relative expression of two genes in the pairs (26). For example, BRG1 expression was more than BRG2 expression, the BRGP score was scored with 1, the BRGP score was scored 0 otherwise. The established BRGPI score of tumor sample completely based on the relative expression of the gene-pair method avoids the batch effect or bias on measurement platforms and is no need for normalization. Then, 311 BRGPs significantly associated with PFS determined by univariate Cox regression analysis (P<0.05) in the signature-training set (GSE93157) were candidates to develop a personalized immune prognostic model in NSCLC. To make the predictive signature more optimized and practical, we selected four gene pairs with the best predictive performance using multivariate Cox regression. Next, we weighted the score of the selected BRGPs by their respective coefficients to obtain the BRGPI. We then determined the best cut-off value to distribute patients into BRGPI-high or BRGPI-low groups by a time-dependent receiver operating characteristic (ROC) curve at one year in the training cohort (29). The predictive performance of the novel BRGPI for immunotherapy response was evaluated in three independent cohorts using the ROC and Kaplan–Meier survival analyses.

### IHC Analysis

We collected the FFPE samples of 19 patients who received anti-PD-1 immunotherapy to obtain the protein expression values of the chosen four gene pairs in the CICAMS cohort. Expression levels of eight genes were determined *via* the IHC method using an anti-human C-C motif chemokine ligand 2 (CCL2, MCP1) antibody (Cat# 25542-1-AP, Proteintech, USA), an anti-human vascular endothelial growth factor A (VEGFA) antibody (Cat# ab52917, Abcam, USA), an anti-human cyclin dependent kinase 1 (CDK1) antibody (Cat# ab133327, Abcam, USA), an anti-human C-X-C motif chemokine ligand 9 (CXCL9, MIG) antibody (Cat# 22355-1-AP, Proteintech, USA), an anti-human major histocompatibility complex, class II, DO beta (HLA-DOB) antibody (Cat# NBP1-87469, NOVUS, USA), an anti-human LCK proto-oncogene, Src family tyrosine kinase (LCK) antibody (Cat# ab32149, Abcam, USA), an anti-human interleukin 12A (IL-12A) antibody (Cat# ab131039, Abcam, USA), and an anti-human T-box 21 (TBX21) antibody (Cat# ab150440, Abcam, USA). Importantly, all IHC slides were assessed based on the evaluation method of the previously published study (30–33). Representative staining images of eight genes from the BRGPI model are shown in **Supplementary Figure S2**.

### Statistical Analysis

Data analysis was performed using GraphPad Prism software (version 5.0) and R software (version 3.6.0).

Survival was assessed using the log-rank test and Kaplan-Meier analysis. Differences between the two groups were evaluated using Chi-square or Mann–Whitney U test. Notably, all statistical analyses were double-sided, and statistical significance was defined as P values less than 0.05.

## Results

### Establishment and Definition of the BRGPI in the Training Cohort

**Figure 1:**
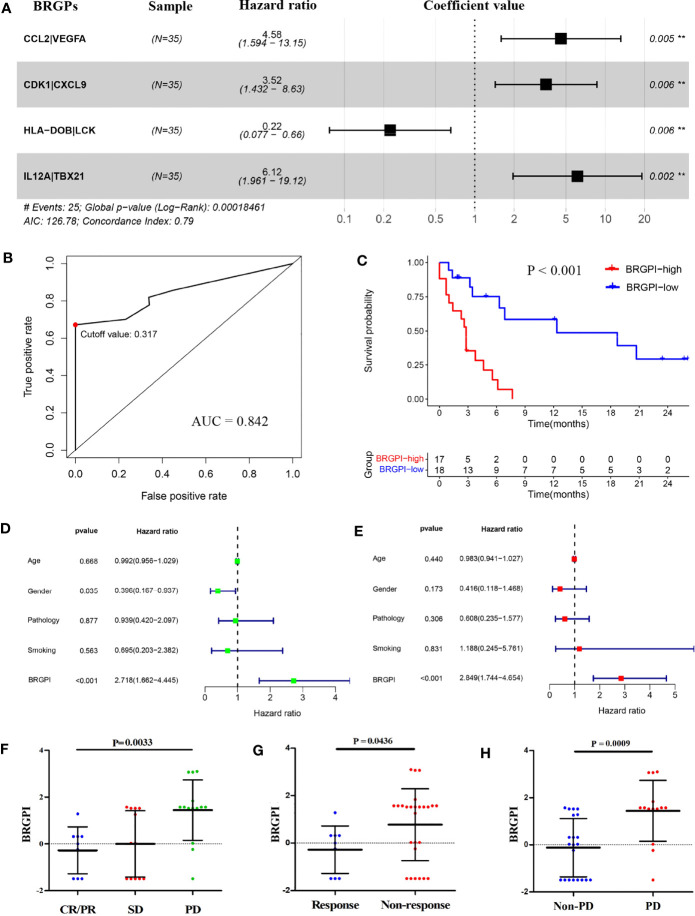
Construction and definition of the BRGPI for patients with NSCLC treated with anti-PD-1 immunotherapy in the training cohort. **(A)** Prognostic values of four selected BRGPs. **(B)** ROC analysis of the BRGPI for progression-free survival. **(C)** Survival curve of progression-free survival for patients with NSCLC treated with anti-PD-1 immunotherapy according to the BRGPI. **(D, E)** Univariate **(D)** and multivariate **(E)** regression analyses of the associations between BRGPI and clinical variables for the predictive ability of progression-free survival. **(F)** The distributions of the BRGPI scores among the patients receiving CR/PR, SD, and PD. **(G)** The distributions of the BRGPI scores between the two groups (response and non-response). **(H)** The distributions of the BRGPI scores between the two groups (Non-PD and PD). **P < 0.01.

To develop a signature to predict patients who might benefit from ICIs, we selected 222 immune-related genes shared by all cohorts and constructed 2526 immune-related gene pairs by pairwise comparison. Next, 311 prognostic BRGPs that were significantly related with PFS (P<0.05) were chosen *via* the univariate Cox proportional hazards regression modelling. We then used multivariate Cox regression to determine gene pairs with the best prognostic performance to obtain the optimized and practical value. According to the minimum criteria, a novel prognostic signature with four BRGPs was proposed (**Figure 1A**). The four selected BRGPs and their coefficients are listed in **Supplementary Table S2**. Next, *via* the multivariate Cox regression, the BRGPI for each patient was scored based on the following formula (33): BRGPI score= 1.521 × value of CCL2|VEGFA +1.257 × value of CDK1|CXCL9 −1.495 × value of HLA-DOB|LCK +1.812 × value of IL-12A|TBX21. According to the optimal cut-off value of 0.317, we classified patients into the BRGPI-low (n=18) and BRGPI-high groups (n=17).

Furthermore, we calculated the AUC value of the ROC and performed Kaplan–Meier survival analysis to validate the predictive performance of the novel BRGPI. The results showed that the AUC value at one-year PFS was 0.842 (**Figure 1B**). Patients with high BRGPI had significantly worse PFS than those with low BRGPI (P<0.001; **Figure 1C**). Next, univariate and multivariate Cox regression analyses were conducted in the training cohort and results showed that BRGPI was an independent prognostic factor (BRGPI: P<0.001, **Figures 1D, E**). We also analyzed the distributions of the BRGPI among the patient subgroups with a different response to immunotherapy. These results showed that patients had a better response in the BRGPI-low group. Furthermore—regardless of the evaluation criteria of the response group—the BRGPI was higher in patients with worse immunotherapy responses, which supports the prediction value of the index (CR/PR, SD, and PD, P=0.0033, **Figure 1F**; response and non-response, P=0.0436, **Figure 1G**; PD and non-PD, P=0.0009, **Figure 1H**). Overall, the predictive ability of the BRGPI for the clinical response of immunotherapy in patients with NSCLC is initially verified and expected to carry next research.

### External Validation of the BRGPI in the Test Cohort

**Figure 2:**
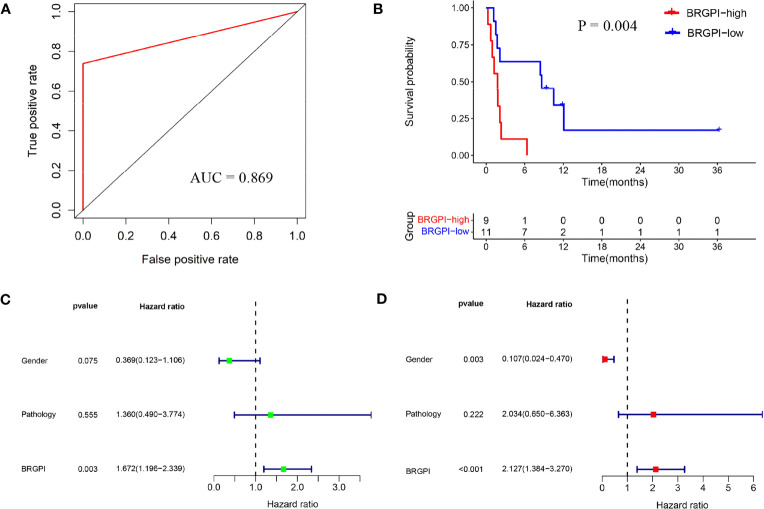
External validation of the BRGPI for patients with NSCLC treated with anti-PD-1 immunotherapy in the test cohort. **(A)** ROC analysis of the BRGPI for progression-free survival. **(B)** Survival curve of progression-free survival for patients with NSCLC treated with anti-PD-1 immunotherapy according to the BRGPI. **(C, D)** Univariate **(C)** and multivariate **(D)** regression analyses of the associations between BRGPI and clinical variables for the predictive ability of progression-free survival.

To confirm the prediction power of BRGPI for anti-PD-1 immunotherapy in NSCLC, we used the same formula for the data in the testing dataset from the GSE136961 cohort. The index of each patient in the GSE136961 cohort was performed and then 20 patients were assigned to the BRGPI-low group (n=11) and BRGPI-high group (n=9) according to the training cohort’s cut-off value. By constructing a ROC curve, the AUC value at a progression-free survival was 0.869. This demonstrated BRGPI had an accurate predictive value for patient prognosis in the testing dataset (**Figure 2A**). *Via* the Kaplan–Meier survival analysis, the results showed that patients with the low-BRGPI score had prominently better PFS than those with the high-BRGPI score (P=0.004; **Figure 2B**). Consistent with the previous findings, univariate and multivariate Cox regression analyses indicated that BRGPI was an independent prognostic factor after adjustment by sex and pathology (BRGPI: P=0.003, **Figure 2C**; BRGPI: P<0.001, **Figure 2D**).

### Independent Validation of the BRGPI in the CICAMS Cohort

**Figure 3:**
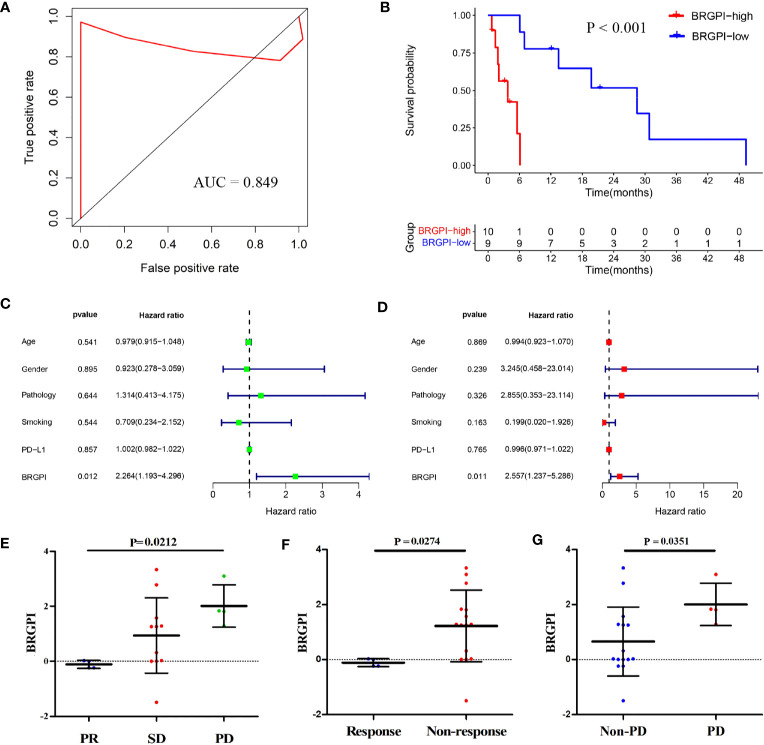
Independent validation of the BRGPI for patients with NSCLC treated with anti-PD-1 immunotherapy in the CICAMS cohort. **(A)** ROC analysis of the BRGPI for progression-free survival. **(B)** Survival curve of progression-free survival for patients with NSCLC treated with anti-PD-1 immunotherapy according to the BRGPI. **(C, D)** Univariate **(C)** and multivariate **(D)** regression analyses of the associations between BRGPI and clinical variables for the predictive ability of progression-free survival. **(E)** The distributions of the BRGPI scores among the patients receiving PR, SD, and PD. **(F)** The distributions of the BRGPI scores between the two groups (response and non-response). **(G)** The distributions of the BRGPI scores between the two groups (Non-PD and PD).

To further access the robustness and practicability of BRGPI, we used protein expression values to investigate its prognostic power in an independent cohort consisting of 19 patients with NSCLC. For each sample, pairwise comparisons for the protein expression values of 8 genes were performed to acquire a score (0 or 1) for each gene pair. We then calculated the BRGPI score of each patient using the mentioned above formula. Representative staining images of eight genes from the BRGPI model are shown in **Supplementary Figure S2**. Given that the AUC value for one year of PFS was 0.849, the BRGPI for patients with NSCLC who received ICIs was a reliable predictive signature at the protein level (**Figure 3A**). We then stratified the 19 patients into a BRGPI-low-group (n=9) and a BRGPI-high group (n=10) with the same cut-off value. The results revealed a notable difference in PFS between the two groups *via* the Kaplan–Meier survival analysis (P<0.001; **Figure 3B**). Consistent with the prior results, univariate and multivariate Cox regression analysis results show that BRGPI was an independent prognostic factor of anti-PD-1 immunotherapy (BRGPI: P=0.012, **Figure 3C**; BRGPI: P=0.011, **Figure 3D**). Further, BRGPI of the CICAMS cohort also can stratify clinically defined groups of patients with different responses (PR, SD, and PD, P=0.0212, **Figure 3E**; response and non-response, P=0.0274, **Figure 3F**; PD and non-PD, P=0.0351, **Figure 3G**), which support the clinical practice value of the prognostic signature.

### Stratification Analysis of BRGPI for Its Predictive Value

To verify the reliability of the BRGPI considering pathology for NSCLC, we performed Kaplan–Meier survival analysis in patients grouped by pathological type for each of the three independent cohorts. Notably, the BRGPI remained highly prognostic for the immunotherapy outcome. In the multicohort analysis—in patients with both non-squamous and squamous-cell NSCLC who were treated with anti-PD-1 immunotherapy—those in the BRGPI-low groups had better PFS than those in the BRGPI-high groups (non-squamous tumors in GSE93157: P<0.001, **Supplementary Figure S3A**; squamous tumors in GSE93157: P=0.032, **Supplementary Figure S3B**; non-squamous tumors in GSE136961: P=0.016, **Supplementary Figure S3C**; squamous tumors in GSE136961: P=0.088, **Supplementary Figure S3D**; non-squamous tumors in CICAMS: P=0.013, **Supplementary Figure S3E**; squamous tumors in CICAMS: P=0.016, **Supplementary Figure S3F**). Noticeably, the statistical significance of Kaplan–Meier survival analysis in squamous tumors from GSE136961 was not significant, but the Kaplan–Meier survival curves of the two groups were slightly separated owning to the very small sample size.

### Association of BRGPI and PD-L1

Given the widespread use of PD-L1 expression level on the cell surface as a validated prediction marker for the response of ICIs, we supposed that BRGPI could improve the prognostic value in combination with the corresponding PD-L1 expression level, although PD-L1 was not a prognostic risk factor in multivariate analyses of CICAMS cohort. Therefore, the prognostic performance of PD-L1 was first assessed *via* the ROC and Kaplan–Meier survival analyses. The AUC value of PD-L1 at PFS was 0.579 for the CICAMS cohort (**Supplementary Figure S4A**). Also, Kaplan–Meier survival analyses did not show a significant difference in PFS of patients with high-expression (n=7) and low-expression (n=12) PD-L1 (**Supplementary Figure S4B**). Nonetheless, the results of the Kaplan–Meier survival analyses the in subset grouped by expression of PD-L1 show that regardless of the expression level of PD-L1, patients in the BRGPI-low group demonstrated longer PFS (P<0.05; **Supplementary Figures S4C, D**), which highlighted the reliable predictive ability of the novel BRGPI. Next, we classified the patients into three subgroups according to the BRGPI and expression level of PD-L1: the BRGPI-low and PD-L1-high group, the BRGPI-low or PD-L1-high group, and the BRGPI-high and PD-L1-low group. As expected, the patients in BRGPI-low and PD-L1-high subgroup derived more clinical benefit while the BRGPI-high and PD-L1-low subgroups derived less clinical benefit (P=0.047; **Supplementary Figure S4E**).

## Discussion

Immunotherapy is revolutionizing cancer treatment, including NSCLC treatment. There has been a rapid rise in the number of ICIs targeting the PD-1/PD-L1 axis clinical trials in NSCLC over the past 15 years. However, it is not effective for all patients. Only a subset will demonstrate durable responses and improved survival after receiving ICI treatment. Although biomarker-related responses to ICI therapy for patients with NSCLC holds promise, there are very few studies within medical literature. Numerous prognostic factors of NSCLC have been continually reported such as PD-L1 expression level and TMB. Currently, detection of PD-L1 expression level is still the standard means of identifying which patients are more likely to benefit from immunotherapy. While owing to different platforms and various cut-off points for the expression between different immunotherapy agents, PD-L1 remains a controversial biomarker for immunotherapy response. In addition, TMB also faces a similar situation as PD-L1. Data across platforms cause biases and the cut-off points may not be reproducible. Therefore, there is an urgent and unmet need for a feasible tool—immune to data source bias—for identifying patients who might derive benefit from anti-PD-1 immunotherapy in clinical practice.

Recent studies show that the immunologic gene expression is correlated with the response to immunotherapy (34). Immunogenic genes related to tumor antigen presentation, chemokine expression, and cytotoxic activity. These features were sufficient for the immunologic landscape (35, 36). A better presentation of the tumor immunologic microenvironment could help identify reliable biomarkers for immunotherapy. The relative ranking of paired-gene expressions provides new ideas for avoiding data preprocessing, such as normalization and scaling. The established BRGPI of the tumor sample, completely based on the relative expression of the gene-pairs method, avoids the batch effect or bias on measurement platforms; there is no need for normalization. The immune-related gene-pair model appears promising for predicting immunotherapy response. Here, we constructed a prognostic BRGPI based on the relative ranking of gene expression values.

In this study, 222 shared immune-related genes from Pan-Cancer Immune Profiling Panel were selected to construct 2526 BRGPs. Then, 311 BRGPs significantly associated with PFS were determined by univariate Cox regression analysis in the signature-training set (GSE93157) and four BRGPs were selected using multivariate Cox regression to calculate the BRGPI. Remarkably, BRGPI can act as an independent prognostic factor and help identify patients in different response groups. By external validation, the GSE136961 cohort also supports the predictive value of BRGPI. Moreover, we further validated the discriminatory performance of BRGPI using protein expression values, acquired using the IHC technique, in an independent CICAMS cohort. The IHC method might be more suitable and convenient for clinical application because of its simplicity and low cost. Considering pathological type—whether non-squamous or squamous-cell NSCLC—patients in BRGPI-low groups had better PFS times than those in the BRGPI-high groups. This indicated that the BRGPI signature is promising preliminary value. We also investigated the association of BRGPI and PD-L1 expression. The predictive ability of PD-L1 was poor in the analyses of the CICAMS cohort. This might be because multiple immune-related genes may better represent the complex immune microenvironment. When patients were grouped by the PD-L1 expression, we found that—no matter PD-L1 expression level—the BRGPI-low subgroup showed longer PFS. Further analysis demonstrated that the patients in BRGPI-low and PD-L1-high subgroup derived more clinical benefit while the BRGPI-high and PD-L1-low subgroups derived less clinical benefit. The combination with PD-L1 underscores the reliability and predictive validity for predicting immunotherapy response, in addition to clinical utility.

BRGPI was constructed by pairwise comparison and the score of each patient was calculated based on his or her own corresponding gene expression. Thus, our prognostic model can avoid the batch effect or bias inherent to different measurement platforms. Additionally, there is no need for data normalization. According to these advantages—and considering the same formula and cut-off value in the training set—this method can be translated into clinical practice as a tool for predicting a patient with NSCLC’s response to immunotherapy.

Nevertheless, the limitations of this study should be acknowledged. First, the size of the three datasets was relatively small, despite our attempts to enroll as many datasets as possible, and inclusion of the GEO and CICAMS cohorts increase the rigor of our biomarker validation process. Second, because this was a retrospective study, further validation of this signature should be conducted in prospective paradigms.

In conclusion, this study was the first to highlight a BRGPI based on benefit-related gene pairs. This method may emerge as a powerful prognostic tool for immunotherapy and help further optimize the ICI paradigm of personalized medicine for patients with advanced NSCLC.

## Data Availability Statement

The datasets presented in this study can be found in online repositories. The names of the repository/repositories and accession number(s) can be found in the article/**Supplementary Material**.

## Ethics Statement 

The Ethics Committee of CICAMS approved the human tissue study protocol, and the approval number was 20/242-2438. The patients/participants provided their written informed consent to participate in this study.

## Author Contributions

CL and SW designed the study, performed experiments, analyzed data, and wrote the manuscript. SZ, FX, ZC, XF, YW, and XQ performed experiments and analyzed data. JH and NS conceived and designed the study and wrote the manuscript. All authors contributed to the article and approved the submitted version.

## Funding

This work was supported by the National Key Basic Research Development Plan (grant number 2018YFC1312105), the National Natural Science Foundation of China (grant numbers 81802299, 81502514), the Graduate Innovation Funds of Peking Union Medical College (grant number 2019-1002-06), the National Key R&D Program of China (grant numbers 2018YFC1312100, 2018YFC1312102), the CAMS Innovation Fund for Medical Sciences (grant numbers 2016-I2M-1-001, 2017-I2M-1-005), and the Fundamental Research Funds for the Central Universities (grant number 3332018070).

## Conflict of Interest

The authors declare that the research was conducted in the absence of any commercial or financial relationships that could be construed as a potential conflict of interest.

## Publisher’s Note

All claims expressed in this article are solely those of the authors and do not necessarily represent those of their affiliated organizations, or those of the publisher, the editors and the reviewers. Any product that may be evaluated in this article, or claim that may be made by its manufacturer, is not guaranteed or endorsed by the publisher.

## Supplementary Material

Supplementary Table 1 Demographic characteristics of NSCLC patients treated with anti-PD-1 immunotherapy in three cohorts.

Supplementary Table 2 Model information about BRGPI.

Supplementary Figure 1 The analysis pipeline of the construction and validation of BRGPI for NSCLC patients treated with anti-PD-1 immunotherapy.

Supplementary Figure 2 Representative staining images of eight genes from the BRGPI model and PD-L1 at different levels.

Supplementary Figure 3 Stratification analysis of BRGPI for its predictive value of progression-free survival in NSCLC patients treated with anti-PD-1 immunotherapy. **(A, B)** Kaplan-Meier survival curve of progression-free survival for non-squamous **(A)** and squamous-cell **(B)** NSCLC patients treated with anti-PD-1 immunotherapy based on the BRGPI in the GSE93157 cohort. **(C, D)** Kaplan-Meier survival curve of progression-free survival for non-squamous **(C)** and squamous-cell **(D)** NSCLC patients treated with anti-PD-1 immunotherapy based on the BRGPI in the GSE136961 cohort. **(E, F)** Kaplan-Meier survival curve of progression-free survival for non-squamous **(E)** and squamous-cell **(F)** NSCLC patients treated with anti-PD-1 immunotherapy based on the BRGPI in the CICAMS cohort.

Supplementary Figure 4 Association of BRGPI and PD-L1 for NSCLC patients treated with anti-PD-1 immunotherapy in the CICAMS cohort. **(A)** ROC analysis of PD-L1 expression for progression-free survival. **(B)** Kaplan-Meier survival curve of progression-free survival for NSCLC patients treated with anti-PD-1 immunotherapy classified by PD-L1 status. **(C, D)** Kaplan-Meier survival curve of progression-free survival for NSCLC patients with **(C)** and without **(D)** positive PD-L1 expression based on the BRGPI after anti-PD-1 immunotherapy. **(E)** Kaplan-Meier survival curve of progression-free survival for NSCLC patients treated with anti-PD-1 immunotherapy among subgroups categorized by BRGPI and PD-L1.
