# Supplementary material for: Avoiding Absolute Quantification Trap: A Novel Predictive Signature of Clinical Benefit to Anti-PD-1 Immunotherapy in Non-Small Cell Lung Cancer
Source: Front Immunol. 2021 Nov 19;12:782106. doi: 10.3389/fimmu.2021.782106 (PMC8640493; doi:10.3389/fimmu.2021.782106)
Supplement: Supplementary Table 1 — Demographic characteristics of NSCLC patients treated with anti-PD-1 immunotherapy in three cohorts. [file Table_1.docx]

| Characteristics | GSE93157 | GSE136961 | CICAMS |
| --- | --- | --- | --- |
| Case No.  Gender, n (%) | 35 | 20 | 19 |
| Male | 27 (77.1) | 15 (75.0) | 14 (73.7) |
| Female | 8 (22.9) | 5 (25.0) | 5 (26.3) |
| Age, n (%) |  |  |  |
| ＜60 | 18 (51.4) | - | 8 (42.1) |
| ≥60 | 17 (48.6) | - | 11 (57.9) |
| Pathology, n (%) |  |  |  |
| Non-squamous | 22 (62.9) | 12 (60.0) | 10 (52.6) |
| Squamous | 13 (37.1) | 8 (40.0） | 9 (47.4) |
| Smoking Status, n (%) |  |  |  |
| Never | 3 (8.6) | - | 7 (36.8) |
| Ever | 32 (91.4） | - | 12 (63.2) |
