# Supplementary material for: Avoiding Absolute Quantification Trap: A Novel Predictive Signature of Clinical Benefit to Anti-PD-1 Immunotherapy in Non-Small Cell Lung Cancer
Source: Front Immunol. 2021 Nov 19;12:782106. doi: 10.3389/fimmu.2021.782106 (PMC8640493; doi:10.3389/fimmu.2021.782106)
Supplement: Supplementary Table 2 — Model information about BRGPI. [file Table_2.docx]

| BRG1 | Full name | Immune processes | BRG2 | Full name | Immune processes | Coefficient |
| --- | --- | --- | --- | --- | --- | --- |
| CCL2 | C-C motif chemokine ligand 2 | Chemokines and receptors; Humoral immune response | VEGFA | vascular endothelial growth factor A | Leukocyte migration;  Cytokines and receptors | 1.521 |
| CDK1 | Cyclin dependent kinase 1 | Innate immune response | CXCL9 | C-X-C motif chemokine ligand 9 | Adaptive immune response; Chemokines and receptors; Regulation of inflammatory response; Th1 orientation | 1.257 |
| HLA-DOB | Major histocompatibility complex, class II, DO beta | Adaptive immune response; Antigen processing and presentation; Cell Type specific; Cytokines and receptors | LCK | LCK proto-oncogene, Src family tyrosine kinase | Regulators of T-cell activation | -1.495 |
| IL-12A | Interleukin 12A | Adaptive immune response; Anti-inflammatory cytokines; Interleukins; NK cell activation; Regulators of Th1 and Th2 development; Th1 orientation | TBX21 | T-box 21 | Adaptive immune response;  Cell Type specific; Regulation of immune response; Th1 orientation | 1.812 |
