# Supplementary figures and images for: Avoiding Absolute Quantification Trap: A Novel Predictive Signature of Clinical Benefit to Anti-PD-1 Immunotherapy in Non-Small Cell Lung Cancer
Source: Front Immunol. 2021 Nov 19;12:782106. doi: 10.3389/fimmu.2021.782106 (PMC8640493; doi:10.3389/fimmu.2021.782106)

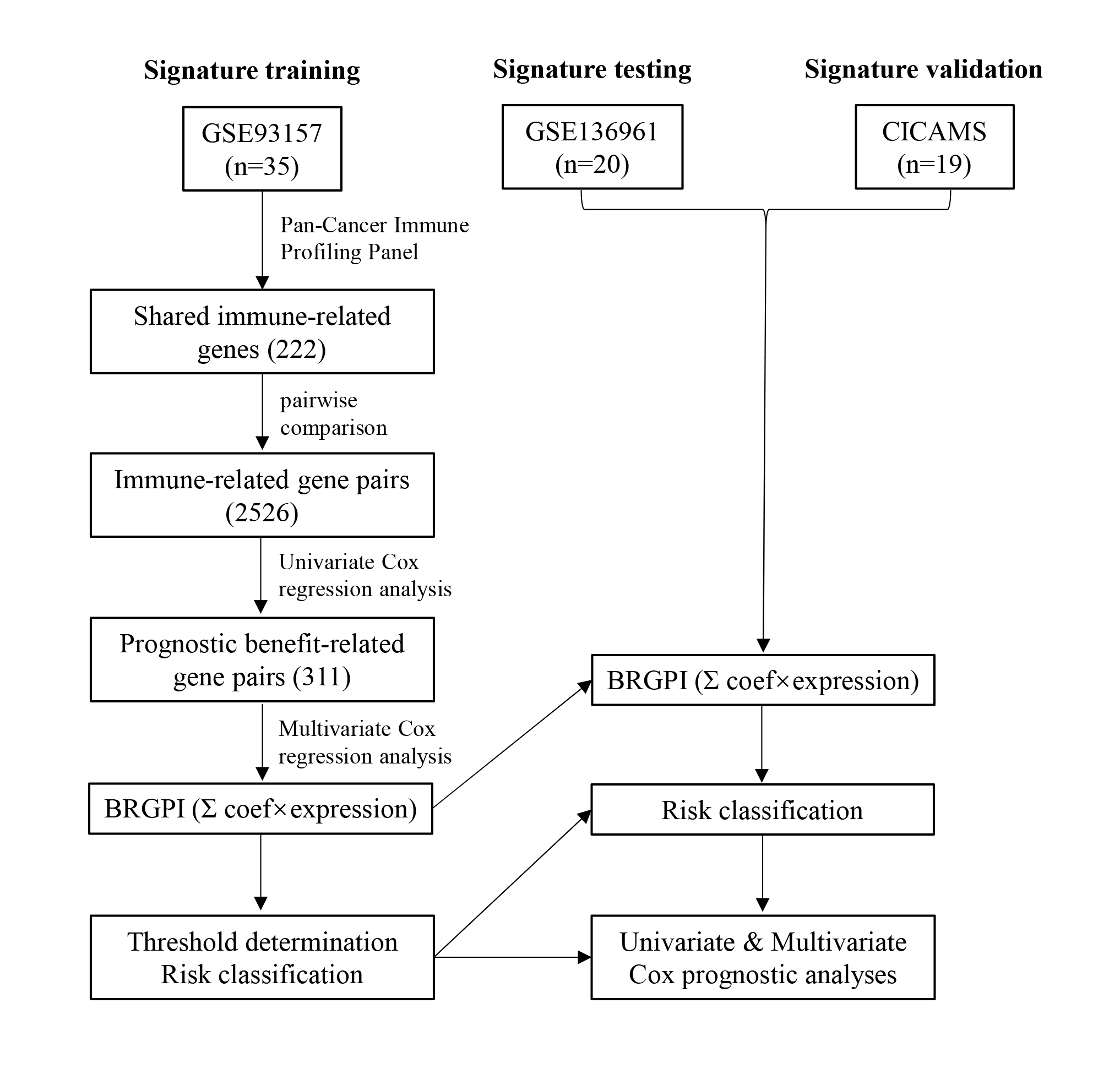

Supplement: Supplementary Figure 1 — The analysis pipeline of the construction and validation of BRGPI for NSCLC patients treated with anti-PD-1 immunotherapy. [file Image_1.tif]

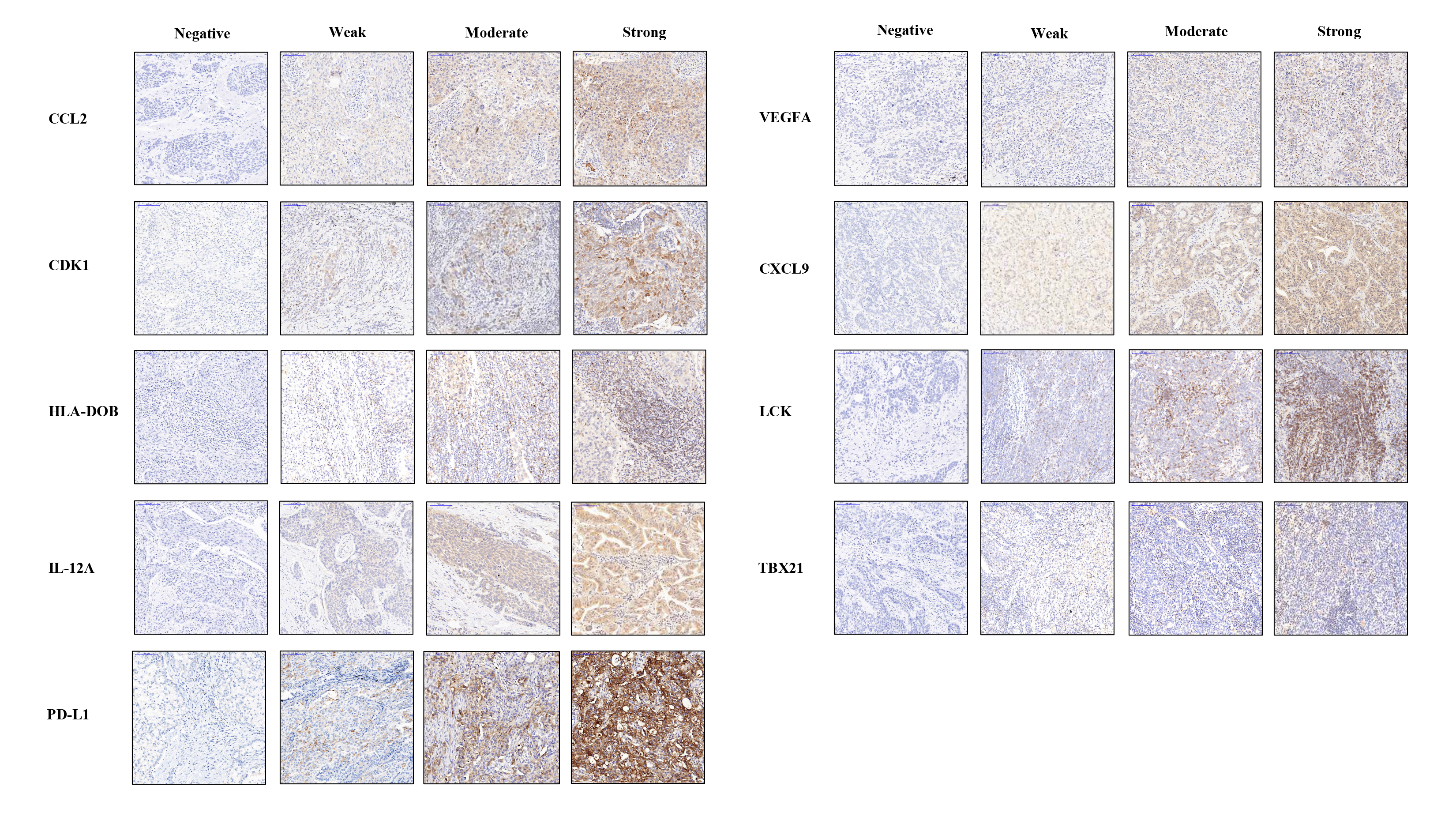

Supplement: Supplementary Figure 2 — Representative staining images of eight genes from the BRGPI model and PD-L1 at different levels. [file Image_2.tif]

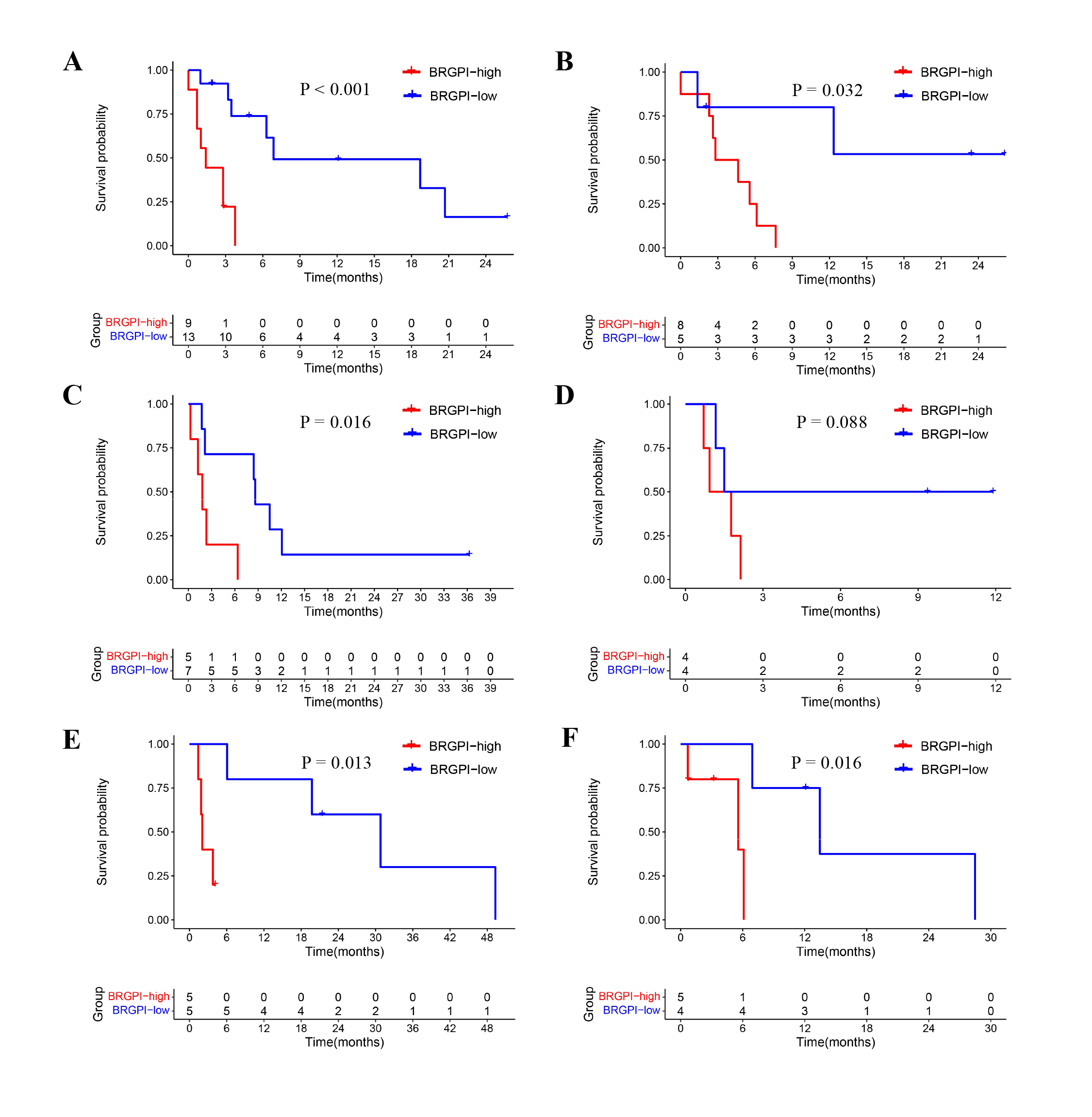

Supplement: Supplementary Figure 3 — Stratification analysis of BRGPI for its predictive value of progression-free survival in NSCLC patients treated with anti-PD-1 immunotherapy. (A, B) Kaplan-Meier survival curve of progression-free survival for non-squamous (A) and squamous-cell (B) NSCLC patients treated with anti-PD-1 immunotherapy based on the BRGPI in the GSE93157 cohort. (C, D) Kaplan-Meier survival curve of progression-free survival for non-squamous (C) and squamous-cell (D) NSCLC patients treated with anti-PD-1 immunotherapy based on the BRGPI in the GSE136961 cohort. (E, F) Kaplan-Meier survival curve of progression-free survival for non-squamous (E) and squamous-cell (F) NSCLC patients treated with anti-PD-1 immunotherapy based on the BRGPI in the CICAMS cohort. [file Image_3.tif]

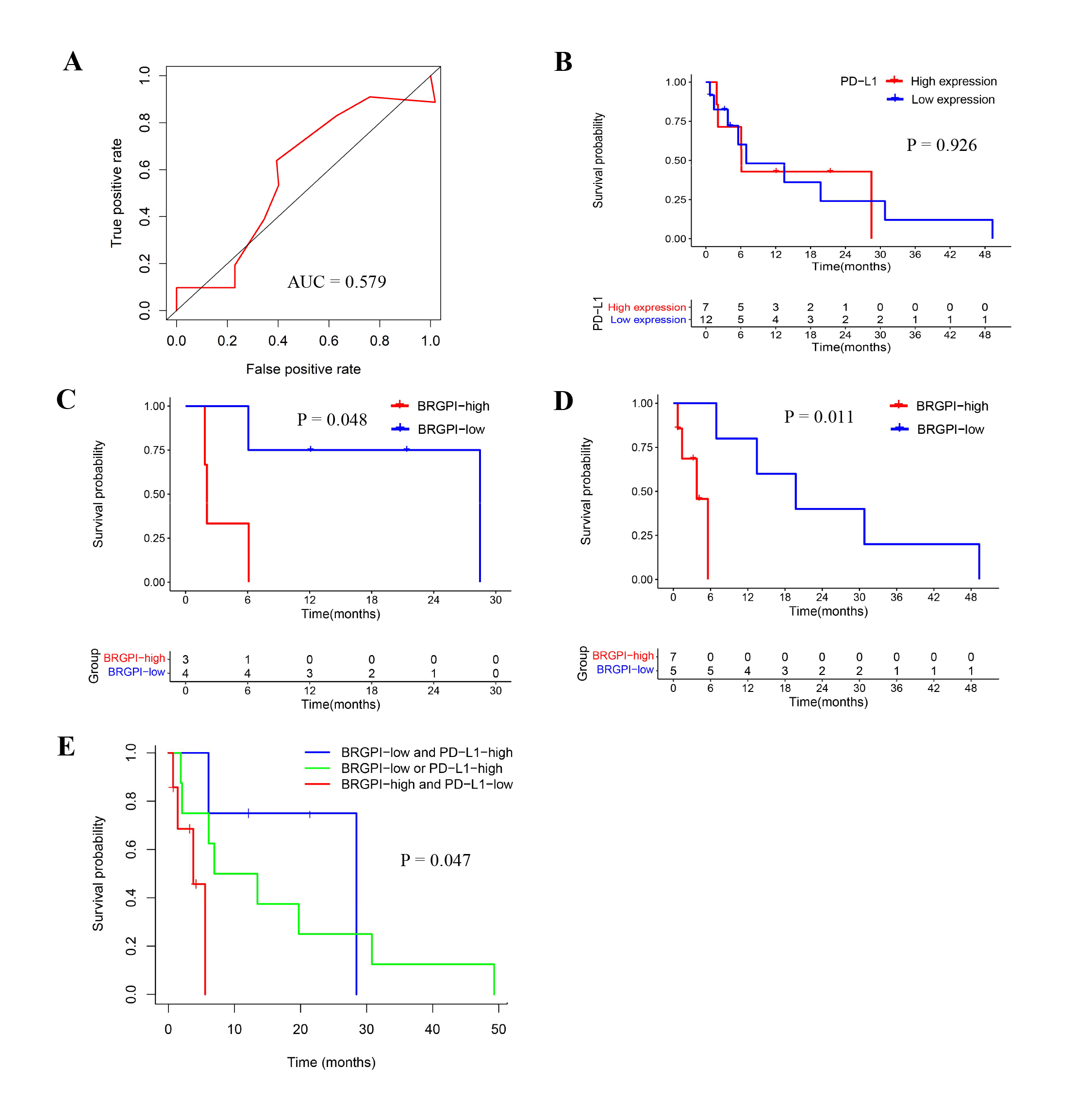

Supplement: Supplementary Figure 4 — Association of BRGPI and PD-L1 for NSCLC patients treated with anti-PD-1 immunotherapy in the CICAMS cohort. (A) ROC analysis of PD-L1 expression for progression-free survival. (B) Kaplan-Meier survival curve of progression-free survival for NSCLC patients treated with anti-PD-1 immunotherapy classified by PD-L1 status. (C, D) Kaplan-Meier survival curve of progression-free survival for NSCLC patients with (C) and without (D) positive PD-L1 expression based on the BRGPI after anti-PD-1 immunotherapy. (E) Kaplan-Meier survival curve of progression-free survival for NSCLC patients treated with anti-PD-1 immunotherapy among subgroups categorized by BRGPI and PD-L1. [file Image_4.tif]
